# The efficacy of a transdiagnostic group cognitive behavioral intervention for Chinese elderly with emotional disorders: A one-year follow-up randomized clinical trial

**DOI:** 10.3389/fpsyt.2022.1027994

**Published:** 2022-11-25

**Authors:** Zijun Yan, Fanqiang Meng, Meiling He, Zhanjiang Li

**Affiliations:** ^1^The National Clinical Research Center for Mental Disorders & Beijing Key Laboratory of Mental Disorders, Beijing Anding Hospital, Capital Medical University, Beijing, China; ^2^Advanced Innovation Center for Human Brain Protection, Capital Medical University, Beijing, China

**Keywords:** transdiagnostic, cognitive behavioral therapy, group therapy, elderly, emotional disorder

## Abstract

**Background:**

With the global aging, geriatric emotional disorders have received more and more attention. Psychotherapy is an effective approach for alleviating the symptoms associated with emotional disorder, but the number of experienced therapists is low. Studies should be conducted to explore a low-cost and efficient treatment method. Previous findings indicate that transdiagnostic cognitive behavior therapy is an effective approach for treatment of emotional disorders. Group therapy is appropriate for the elderly as they are characterized by high levels of loneliness. In this study, we compared and explored the effects of a transdiagnostic group cognitive behavioral intervention (TD-GCBT), a transdiagnostic individual cognitive behavioral intervention (TD-CBT), and treatment as usual (TAU) on treatment of emotional disorders among the elderly.

**Method:**

A total of 120 elderly patients diagnosed with emotional disorders were randomly assigned to the TD-GCBT group (40), TD-CBT group (40), and TAU group (40). Changes in symptoms were assessed using HAMD, PHQ-9, HAMA, and GAD-7 scales at baseline, post-treatment (three months), six-month and twelve-months follow-up. The efficacies of the three intervention strategies were compared using linear mixed-effects models. *Post-hoc* and simple effect analyses were conducted to determine the differences among the three groups.

**Results:**

The HAMD, PHQ-9, HAMA, and GAD-7 scores revealed a significant effect from baseline to 12 months for time (*p* < 0.001), group (*p* < 0.001) and time × group interaction (*p* < 0.001) in TD-GCBT group compared with the TD-CBT group and TAU group. The effect of TD-GCBT (HAMD: Cohen’s *d* (3th month, 6th month, 12th month) = 2.69, 3.98, 4.51; HAMA: Cohen’s *d* = 2.84, 4.13, 5.20) and TD-CBT (HAMD: Cohen’s *d* = 2.55, 2.87, 2.63; HAMA: Cohen’s *d* = 2.43, 2.83, 2.78) group was better relative to that of the TAU group (HAMD: Cohen’s *d* = 0.41, 1.13, 1.46; HAMA: Cohen’s *d* = 0.64, 1.22, 1.57) (*p* < 0.001). The scores of the TD-GCBT group showed the most significant decrease compared with the other two groups.

**Conclusion:**

The findings indicate that TD-GCBT method is effective for treatment of emotional disorders among the elderly. TD-GCBT is effective for alleviating depression and anxiety symptoms up to at least nine months after treatment. The results indicate that TD-GCBT is a cost-effective and resource-effective strategy and can be used an alternative therapy for treatment of mental disorders.

**Clinical trial registration:**

[https://www.chictr.org.cn], identifier [ChiCTR1900021806].

## Introduction

The global aging population has markedly increased in the recent years due to increase in life expectancy and decreased fertility (1). The seventh national census released on 11 November 2021 indicated that 260 million people are aged 60 and above in China, accounting for about 20% of the total population (2). Various physical health conditions increase the risk of developing mental health disorders among the elderly (3, 4). Emotional disorders, including anxiety, depression, fear, and somatic symptoms, are the most common mental disorders (5). These emotional disorders have varying symptoms, including fear, anxiety, depression, or physical components (6). In addition, these disorders are characterized by common cognitive distortions, such as neglecting the role of positive factors, negative thoughts, and pessimistic explanatory style (7–9). Depression and anxiety disorders are the most prevalent mental disorders among adults aged 60 and above. Depression and anxiety disorders affect about 7 and 3.8% of the world’s elderly population, respectively (10). Comorbidity of anxiety and depression among the elderly is more common than occurrence of each disorder independently (11). A study by Lenze reported a high rate of lifetime (35%) and current (23%) comorbid anxiety disorders (12). Depression and anxiety comorbidity is associated with severe emotional symptoms, higher risk of suicide, higher impairment of social functioning, greater memory decline and more somatic complaints compared with occurrence of the either of the disorders (4). These severe symptoms can significantly affect subjective well-being and quality of life of the patients (13). Only less than 50% of patients receive the appropriate treatment despite the high prevalence and negative impact of emotional disorders among the elderly (14).

Pharmacotherapy is a common treatment approach for elderly patients with emotional disorders (15, 16). However, pharmacotherapy is associated with slow response to treatment, limited remission rate, and high risk of relapse (17). In addition, pharmacotherapy is characterized by adverse side effects and a high risk of interactions with drugs prescribed for comorbid conditions among the elderly (18). Psychotherapy is an effective strategy for treatment of depression and anxiety disorders among the elderly (19, 20), and the combination of medication and psychotherapy is often used in clinic. Cognitive behavioral therapy (CBT) is the most widely studied psychotherapy due to its high efficacy (21, 22). CBT reduces relapse rates during maintenance treatment (19). However, the strategy requires large number of specialized therapists who should be familiar with the treatment procedures for each disorder. The high incidence of comorbid depression and anxiety in elderly implies that specific disorder prevention interventions may be effective for target disorders, but may not effectively improve other comorbid emotional disorders (23).

Transdiagnostic individual CBT (TD-CBT) is an alternative treatment approach, which is effective for individual and comorbid disorders as it targets common symptoms and factors (24). Marnoch (25) conducted a pilot study to explore the effect of TD-CBT for treatment of elderly patients with comorbid depression and anxiety. The findings showed a significant decrease in the level of depression and anxiety, indicating a potential therapeutic effect of TD-CBT method. Moreover, TD-CBT can concurrently alleviate the symptoms of depression and anxiety, which can benefit more people. It is easier for therapists to master the procedures for TD-CBT than for each specific disorders. Notably, a high number of therapists is still required for treatment of the high number of cases with emotional disorders. Furthermore, the elderly has a poor social support system, most have lost their spouses, or lack care from their children due to work commitments. As a result, current studies are exploring the efficacy of transdiagnostic group cognitive behavioral therapy (TD-GCBT) for treatment of the elderly patients. Patients with similar symptoms can converse and share their experiences. These social interactions can reduce the symptoms and loneliness among the elderly, as well as reduce the high demand for psychotherapy. A previous study was conducted to explore the effect of TD-GCBT for treatment of the elderly with comorbid anxiety and depression, and unipolar mood disorder (26). The findings showed that TD-GCBT effectively alleviated the symptoms of depression and anxiety among the elderly. Follow-up results showed that the TD-GCBT strategy has long-term effects, indicating the therapeutic potential of TD-GCBT.

To the best of our knowledge, no study has been conducted to explore the use of TD-GCBT for treatment of emotional disorders among Chinese elderly. Currently, the number of psychotherapists in China is insufficient to meet the needs of psychotherapy owing to the high prevalence of emotional disorders. Therefore, it is imperative to explore more effective and cost-effective treatment strategies. In the present study, the Delphi method was used in a pilot study to explore more effective treatment technologies for the elderly due to the high dropout rate in previous studies (27). The results showed that supportive strategies, homework, behavior-focused methods such as behavioral activation and relaxation training, as well as emotion-focused approaches such as emotional awareness and recognition techniques are preferred for TD-GCBT among the elderly. Cognitive-focused strategies are more effective in the later stages of treatment. These intervention methods were used and foreign guidelines were reviewed to develop a Chinese version of the TD-GCBT intervention for emotional disorders among the elderly. Moreover, more humanistic care and appropriate support were provided to the elderly patients, to effectively establish relationships among the patients and reduce the drop rate. The aim of this study was to explore the clinical effect and application of the Chinese operation program by comparing TD-GCBT with TD-CBT and TAU strategies. The hypothesis of the study was that the efficacy of TD-GCBT and TD-CBT was similar, and the two strategies were more effective compared with the TAU approach. The findings of this study will provide a reference and practical basis for further development and application of TD-GCBT approach in China.

## Methods

### Participants

This RCT study was conducted at Beijing Anding Hospital in China from February 2018 to August 2020. Eligible patients attending the anxiety or depression disorder clinics were recruited for the study. The inclusion criteria were: (a) Patients aged 60–75 years; (b) Patients diagnosed with depression, generalized anxiety disorder, panic disorder, social anxiety disorder, or comorbidities through a structured clinical interview according to DSM-IV criteria conducted by well-trained psychiatrists; (c) Patients with junior high school education or above; (d) Patients who had been stable for more than a month after taking drugs (Administered drugs were limited to sertraline, citalopram, escitalopram, paroxetine, mirtazapine, venlafaxine, and duloxetine).

Exclusion criteria were: (a) Patients with active suicidal thoughts; (b) Patients with severe/unstable physical illness or neurological disorder; (c) Patients with a current or history of any other psychiatric disorders, including drug or alcohol dependence; (d) Patients who had undergone convulsion-free electroconvulsive therapy (MECT) within three months; (e) Patients with cognitive dysfunction.

**FIGURE 1:**
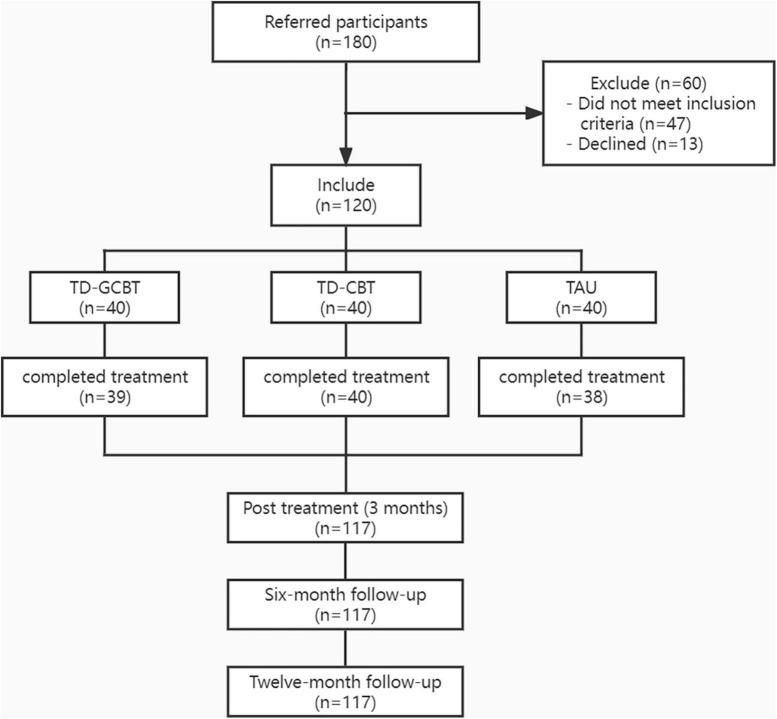
Flow chart of enrollment and randomization.

All participants and their guardians signed an informed consent form. The trial was registered with the China Clinical Trial Registry: ChiCTR1900021806. The design for the trial is presented in Figure 1.

### Sample size

We used the G*Power program. Due to sufficient funding, we set the effect size between groups to 0.25, α to 0.001, and power to 0.99. It was planned to be divided into three groups, and the minimum sample size for each group was 31. According to the dropout rate of 20%, at least 39 samples were needed for each group. Therefore, in this study, there are 40 cases in each group with a total of 120.

### Intervention

#### Transdiagnostic group cognitive behavioral intervention

The TD-GCBT intervention program for emotional disorders among the elderly was established following the Canadian operation manual “The Change ways Geriatric Participant Manual (28)”, which is reported to be effective. The technique suitable for CBT among the transdiagnostic group of emotional disorders in the elderly was determined using the Delphi method by therapists and geriatric psychiatrists (27). CBT combined with routine intervention may achieve a better therapeutic effect.

Each sub-group in the therapy group had 6–8 patients and two therapists. The treatment approaches included therapist guidance and group interaction. The interventions lasted for 12 weeks and comprised eight sessions, with each session lasting 90 min. Therapists held a separate meeting with the patient in case the patient had an emergency. The initial four sessions were conducted once a week, whereas the last four sessions were conducted once every two weeks. Relationship building, theoretical health education and goal setting were covered during the first and second sessions. Identifying helpless thoughts, cognitive reconstruction and worry management were covered in Sessions 3–6. Sleep disorders, sustainable lifestyle, dealing with loneliness and relapse prevention were covered in Sessions 7–8. The subjects were assigned homework before the end of each session, which was checked at the beginning of the next session (Table 1).

**TABLE 1:** TD-GCBT intervention programs.

	Topic	Main contents
Session1	Know each other	Relationship building, group introduction, goal setting
Session2	Normalization	Health education of aging theory and emotional disorders
Session3	Go into action	Cognitive triangle model, thought record, behavioral activation, relaxation
Session4	Challenge oneself	Emotional acceptance practice, downward arrow, identification of distorted thoughts
Session5	Recognize the self	Socratic questioning, cognitive distortions recognition
Session6	Loneliness	Solution to loneliness, behavioral experiment
Session7	Solve the problems	Coping styles and sleep problems
Session8	Relapse prevention	Continue the discussion of sleep problems, relapse prevention

#### Transdiagnostic individual cognitive behavioral intervention

The TD-CBT intervention program was conducted based on the TD-GCBT manual separately for each participant. In this case, one therapist handled one patient at a time. Therapists intervened with emotional disorders using cognitive behavioral techniques suitable for transdiagnosis in older patients. The duration of the intervention was 12 weeks with eight sessions each lasting 60 min. Therapists identified dysfunctional cognitions and targeted emotional disorders using cognitive triad, thinking diary, Socratic questioning and other approaches. The therapists changed the patient behavior using techniques, such as behavioral activation and relaxation training. In addition, the loneliness and sleep disorders among the elderly were explored and resolved.

#### Treatment as usual group

Patients in the TAU group received routine medical interventions. The prescription was given by the psychiatrist based on his/her experience, potential side effects, pharmacokinetic considerations, and patient preference. Sertraline, citalopram, escitalopram, paroxetine, mirtazapine, venlafaxine, and duloxetine were administered to this group.

### Therapists

The therapist group comprised six experienced psychiatric nurses who had psychotherapist qualification. The therapists were assigned to two groups: the leaders and observers. All therapists had received systematic CBT and TD-CBT training for elderly emotional disorders prior to the study. In addition, the therapists conducted three simulation interventions for actual cases before participating in the current study. Each case was supervised at least once at the middle stage of the intervention to ensure that the therapists followed the protocol.

### Supervision

The psychotherapy process was supervised by peer and experts with several years of experience in CBT for the elderly. Peer supervision for the therapists was conducted once a week, and expert supervision was conducted once every 2 weeks through online conference or face-to-face meetings. Therapists submitted written case reports, treatment plans, therapy progress, and questions administered for each case during sessions. Peers and experts participated in solving challenges during the intervention and ensured the process was conducted according to the manual to improve the quality of intervention.

### Measurements

#### Diagnostic measures

All participants completed the structured clinical interview for DSM-IV (SCID-IV) (29) conducted by the psychiatrists. SCID-IV is a semi-structured clinical interview with high reliability. Trained psychiatrists administered the SCID to patients to assess Axis I disorders.

#### Mini-mental state examination

Elderly patients with cognitive impairment were excluded after conducting MMSE (30). The MMSE scale has a total score of 30. The threshold value was limited to 17 points for illiteracy, 20 points for primary school, and 24 points for secondary school and above. The MMSE scores of patients that participated in this study were above 27.

#### Hamilton depression scale

The severity of depressive symptoms was assessed using 17 versions in the last week of the intervention. Well-trained psychiatrists used the HAMD scale to analyze depression symptoms (31). HAMD scale has good reliability and validity in clinical applications. A high total score indicates more severe depressive symptoms.

#### Patient health questionaire-9 items

The PHQ-9 scale (32) was used to identify and evaluate depressive symptoms among participants. The scale comprised scores from 0 (not at all) to 3 (almost daily). The total scores of the PHQ-9 scale ranged from 0 to 27 and were denoted as follows: 0 to 5 indicating no depression, 6 to 9 mild depression, 10 to 14 moderate depression, 15 to 19 severe depression and 20 to 27 extremely severe depression.

#### Hamilton anxiety scale

The severity of anxiety symptoms was assessed using the Chinese version of the HAMA scale (33). The scale comprised 14 items. The scale was used to assess the patients’ condition in the last week of intervention. Each item in this scale is divided into five grades, each evaluated based on a scale of 0 to 4. This scale has good inter-rater reliability and validity. A high score indicates a high degree of anxiety.

#### Generalized anxiety disorder-7 items

Generalized anxiety disorder-7 items (GAD-7) criteria are used to examine generalized anxiety and evaluate the severity of symptoms (34). In addition, it can be used to assess panic disorder and PTSD. The scale comprises 7 items with scores ranging from 0 to 4 to indicate lack of anxiety, 5 to 9 to indicate mild anxiety, 10 to 14 to represent moderate anxiety and 15 to 21 to denote severe anxiety.

### Patient safety

Adverse events were monitored by getting feedback and through observation of participants during treatment and the follow-up period. Therapists took notes and made timely emergency interventions when patients presented with adverse events, such as signs of suicide, taking sub-optimal medication, or increased psychological distress. The patients were referred to the psychiatrist for active management of adverse reactions depending on the severity.

### Statistical analysis

All statistical analyses were conducted using R version 4.0.5. The R packages “lme4” and “emmeans” were used for statistical analysis. One-way ANOVA or chi-squared tests were used to determine the baseline differences of demographic and clinical variables among the three groups. Linear mixed-model analysis was performed to determine the main effects of group, time point and the interaction between group and time point. *Post-hoc* (LSD) and simple effect analysis was conducted for pairwise comparison of the groups. Two-tailed tests were performed in all analyses, with the significance level set at 0.05.

## Results

### Dropout rates

A total of 120 patients were randomly assigned to three intervention groups. One patient in the TD-GCBT group and two patients in the control group dropped out during the intervention. The overall dropout rate was 2.5% (3/120). One person in the TD-GCBT group dropped out during the treatment due to significant alleviation of symptoms. Two patients in the TAU group were lost at the 12th week of follow-up.

### Preliminary analyses

A total of 117 patients completed the entire study. No adverse reactions were reported among the participants. Details on the diagnosis of participants are presented in Table 2. The demographic characteristics and data from the various scales for participants in the experimental and control groups are shown in Table 3. The results showed no significant between-group differences before the intervention.

**TABLE 2:** Diagnostic description.

Diagnosis	TD-GCBT	TD-CBT	Control group	Total
Major depressive disorder	8	4	9	21
Generalized anxiety disorder	14	6	7	27
Panic disorder	0	0	1	1
Social anxiety disorder	0	0	0	0
Major depressive disorder comorbid with Generalized anxiety disorder	15	24	16	55
Major depressive disorder comorbid with Panic disorder	1	1	1	3
Generalized anxiety disorder comorbid with Panic disorder	1	2	2	5
Major depressive disorder comorbid with Social anxiety disorder	0	1	0	1
Panic disorder comorbid with Social anxiety disorder	0	0	1	1
Major depressive disorder comorbid with Generalized anxiety disorder and Panic disorder	0	2	1	3

**TABLE 3:** Comparison of demographic and clinical data among the three groups at baseline.

Variables	TD-GCBT (*n* = 39)	TD-CBT (*n* = 40)	TAU (*n* = 38)	*F*/χ*2*	*p*
Gender				3.932	0.140
Male	7	7	13		
Female	32	33	25		
Age, years	66.31(7.75)	65.22(3.80)	66.50(4.77)	0.575	0.564
Education				6.245	0.620
Junior high school	9	10	12		
Senior high school	14	13	14		
Junior college	6	10	8		
Undergraduate	10	6	4		
Master degree or above	0	1	0		
Living situation				0.223	0.895
Living alone	4	3	3		
Living with family	35	37	35		
Family history				5.490	0.064
No	36	30	34		
Yes	3	10	4		
HAMD	22.97(6.07)	24.95(5.54)	22.89(6.22)	1.515	0.224
PHQ-9	12.13(4.36)	14.08(4.64)	14.45(5.53)	2.550	0.083
HAMA	21.33(3.98)	22.65(3.79)	20.58(5.40)	2.189	0.117
GAD-7	12.38(4.32)	14.33(3.39)	12.47(4.85)	2.663	0.074

HAMD, Hamilton Depression Scale; PHQ-9, Patient Health Questionaire-9 items; HAMA, Hamilton Anxiety Scale; GAD-7, Generalized anxiety disorder-7 items.

### Comparative effects among three groups

#### Primary outcome: change in hamilton depression scale and hamilton anxiety scale scores over time

**FIGURE 2:**
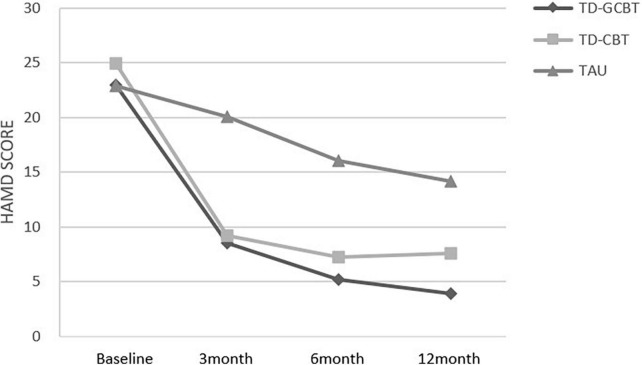
Mean HAMD total scores for the three groups from baseline to 12 months for the three groups.

**FIGURE 3:**
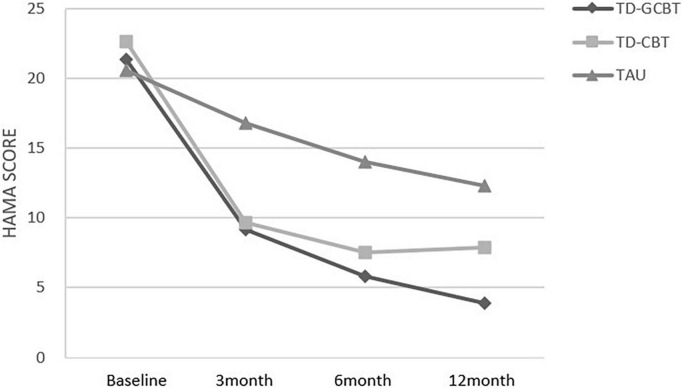
Mean HAMA total scores for the three groups from baseline to 12 months for the three groups.

Linear mixed-effects model analyses of the HAMD and HAMA scores revealed a significant effect of time (*p* < 0.001), group (*p* < 0.001) and time × group interaction from baseline to 12 months (*p* < 0.001; Table 4 and Figures 2, 3). *Post-hoc* (LSD) and simple effect analyses were conducted to further explore the changes and specific pairwise differences between groups (Tables 5, 6). The response rate for participants in the TD-GCBT group based on HAMD (reduction rate > 50%) was 71.79%, whereas that based on the HAMA scale was 66.67%. The response rate of subjects in the TD-CBT group based on HAMD and HAMA scales were 82.5 and 80%, respectively. The response rate of participants in the TAU group based on HAMD was 0 and that based on the HAMA scale was 2.63%. The effect sizes (Cohen’s *d*) of the changes in three groups at different time points are presented in Table 7.

**TABLE 4:** Interaction between group and time among three groups.

Measures	df	*F*	*p*
**HAMD**			
Time	342	333.799	< 0.001
Time*Group	342	24.608	< 0.001
Group	111	27.135	< 0.001
**PHQ-9**			
Time	342	151.468	< 0.001
Time*Group	342	30.065	< 0.001
Group	110	30.065	< 0.001
**HAMA**			
Time	342	378.055	< 0.001
Time*Group	342	21.715	< 0.001
Group	111	15.805	< 0.001
**GAD-7**			
Time	342	378.054	< 0.001
Time*Group	342	21.715	< 0.001
Group	111	15.804	< 0.001

**TABLE 5:** Changes in depression and anxiety from the completion of treatment to the twelve-month follow-up.

Group	Measures	Baseline M(SD)	Post-treatment (3th month) M(SD)	Values (Baseline-3th month)		Six-month follow-up M(SD)	Values (3th month-6th month)		Twelve-month follow-up M(SD)	Values (6th month–12th month)	
				t	*p*		t	*p*		t	*p*
TD-GCBT (*n* = 39)	HAMD	22.97(6.07)	8.51(4.70)	15.581	<0.001	5.15(2.89)	3.619	0.002	3.87(2.40)	1.381	1.000
	PHQ-9	12.13(4.36)	3.97(3.24)	11.505	<0.001	3.10(2.52)	1.230	<0.001	2.28(2.04)	1.158	1.000
	HAMA	21.33(3.98)	9.18(4.59)	15.711	<0.001	5.79(3.55)	4.375	<0.001	3.90(2.72)	2.453	0.088
	GAD-7	12.38(4.32)	4.05(3.05)	12.936	<0.001	2.90(2.35)	1.791	<0.001	1.90(1.89)	1.552	0.729
TD-CBT (*n* = 40)	HAMD	24.95(5.54)	9.23(6.80)	17.158	<0.001	7.20(6.83)	2.210	0.167	7.58(7.66)	–0.409	1.000
	PHQ-9	14.08(4.64)	6.05(4.68)	11.467	<0.001	4.97(5.80)	1.536	0.753	4.47(5.15)	0.714	1.000
	HAMA	22.65(3.79)	9.65(6.920)	17.019	<0.001	7.55(6.89)	2.749	0.038	7.87(6.83)	–0.425	1.000
	GAD-7	14.33(3.39)	6.23(4.35)	12.734	<0.001	4.32(4.31)	2.987	0.018	3.45(4.36)	1.376	1.000
TAU (*n* = 38)	HAMD	22.89(6.22)	20.08(7.45)	2.995	0.018	16.08(5.86)	4.250	<0.001	14.13(5.78)	2.071	0.235
	PHQ-9	14.45(5.53)	12.03(6.16)	3.372	0.005	11.50(5.88)	0.733	1.000	10.53(4.27)	1.356	1.000
	HAMA	20.58(5.40)	16.76(6.60)	4.869	<0.001	14.03(5.35)	3.492	0.003	12.29(5.16)	2.216	0.164
	GAD-7	12.47(4.85)	11.66(6.20)	1.250	1.000	7.97(3.41)	5.645	<0.001	7.29(3.12)	1.048	1.000

**TABLE 6:** Comparison of differences among the three groups at the same time point.

Time	Group	HAMD		PHQ-9		HAMA		GAD-7	
		t	*p*	t	*p*	t	*p*	t	*p*
Baseline	TD-GCBT × TD-CBT	–1.446	0.448	–1.741	0.249	–1.106	0.810	–2.085	0.114
	TD-GCBT × TAU	0.401	1.000	–2.070	0.119	0.691	1.000	–0.163	1.000
	TD-CBT × TAU	1.824	0.208	–0.375	1.000	1.780	0.230	1.884	0.182
Post-treatment (3 months)	TD-GCBT × TD-CBT	–0.486	1.000	–1.864	0.191	–0.405	1.000	–2.347	0.059
	TD-GCBT × TAU	–8.236	<0.001	–7.400	<0.001	–6.036	<0.001	–8.402	<0.001
	TD-CBT × TAU	–7.811	<0.001	–5.617	<0.001	–5.677	<0.001	–6.149	<0.001
Six-month follow-up	TD-GCBT × TD-CBT	–1.500	0.405	–1.670	0.289	–1.469	0.430	–1.509	0.397
	TD-GCBT × TAU	–7.761	<0.001	–7.721	<0.001	–6.559	<0.001	–5.629	<0.001
	TD-CBT × TAU	–6.337	<0.001	–6.130	<0.001	–5.160	<0.001	–4.181	<0.001
Twelve-month follow-up	TD-GCBT × TD-CBT	–2.760	0.019	–1.976	0.148	–3.309	0.003	–1.650	0.301
	TD-GCBT × TAU	–7.268	<0.001	–7.578	<0.001	–6.688	<0.001	–5.975	<0.001
	TD-CBT × TAU	–4.604	<0.001	–5.687	<0.001	–3.486	0.002	–4.392	<0.001

**TABLE 7:** Effect sizes (Cohen’s d) of three groups in four scale scores.

Group	Measures	Post-treatment (3 months)	Six-month follow-up	Twelve-month follow-up
TD-GCBT	HAMD	2.69	3.98	4.51
	PHQ-9	2.15	2.63	3.08
	HAMA	2.84	4.13	5.20
	GAD-7	2.26	2.84	3.38
TD-CBT	HAMD	2.55	2.87	2.63
	PHQ-9	1.72	1.75	1.96
	HAMA	2.43	2.83	2.78
	GAD-7	2.09	2.60	2.81
TAU	HAMD	0.41	1.13	1.46
	PHQ-9	0.41	0.52	0.80
	HAMA	0.64	1.22	1.57
	GAD-7	0.15	1.09	1.30

#### Secondary outcome: change in patient health questionaire-9 items and generalized anxiety disorder-7 items scores over time

**FIGURE 4:**
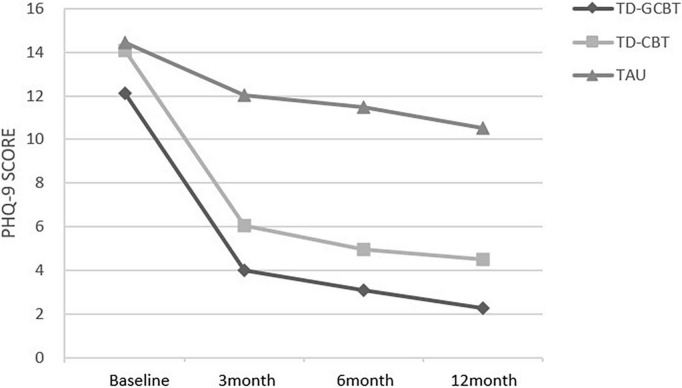
Mean PHQ-9 total scores for the three groups from baseline to 12 months for the three groups.

**FIGURE 5:**
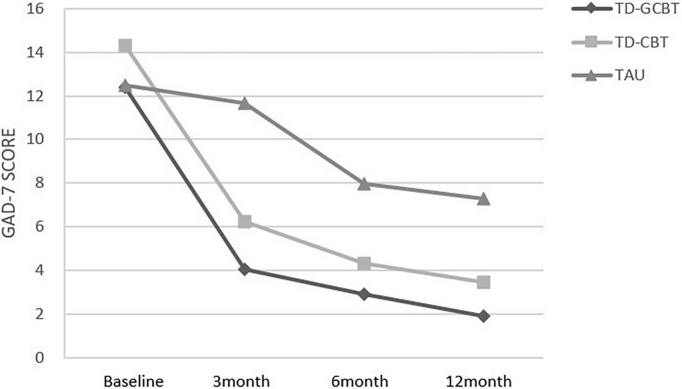
Mean GAD-7 total scores for the three groups from baseline to 12 months for the three groups.

The PHQ-9 and GAD-7 scores revealed a significant main effect of time, group and significant time × group interaction (*p* < 0.001). These results indicate a significant alleviation of depression and anxiety symptoms in the three groups over time (Figures 4, 5).

### Treatment satisfaction

The treatment satisfaction in this study was evaluated by patients reporting whether treatment was effective and whether they were willing to recommend it to other patients. Treatment satisfaction was evaluated based on a scale of 1 (totally disagree) to 5 (totally agree). The mean scores for the TD-GCBT group for treatment efficacy was 4.67 ± 0.530, that of the TD-CBT group was 4.80 ± 0.384, and that of the TAU group was 4.47 ± 0.763. Patients in the TD-CBT group and TAU group showed significant differences (*p* = 0.013) in treatment satisfaction. The mean scores on whether to recommend the treatment to other patients for the TD-GCBT group, TD-CBT group and TAU group were 4.74 ± 0.442, 4.80 ± 0.445 and 4.51 ± 0.384, respectively. The satisfaction scores of the TD-GCBT group and TD-CBT group were significantly higher relative to that of the TAU group (*p* < 0.05). However, there was no significant difference in satisfaction scores between the TD-GCBT group and TD-CBT group (*p* = 0.547).

## Discussion

China is the most populated nation in the world and has the highest elderly population globally (2). Notably, the social support system for the elderly in China is not advanced (35). The elderly has a higher risk for emotional disorders, such as depression and anxiety disorders due to the physical and psychological characteristics. Researchers are currently exploring effective and less costly treatments for elderly subjects diagnosed with emotional disorders. Previous studies report that TD-GCBT is an effective intervention for management of emotional disorders among the elderly (26). In the current study, the baseline and clinical characteristics of participants in the TD-GCBT, TD-CBT, and TAU groups were evaluated. The results showed that TD-GCBT is an effective intervention approach for emotional disorders among elderly Chinese subjects. This finding provides a reference for the use of psychotherapy in more patients as it is highly effective and less costly. Several psychiatric nurses in China have undergone psychotherapy training and have extensive psychotherapy expertise. Therefore, these trained nurses can serve as psychotherapists. The present study is the first RCT study on the use of TD-GCBT for the management of emotional disorders among the elderly in China.

The findings indicated that the depression and anxiety scores of the TD-GCBT group and TD-CBT group significantly decreased after 12 weeks of intervention. The HAMD, HAMA, and PHQ-9 scores in the TAU group were significantly lower after the intervention compared with the baseline scores. These findings indicate that the three intervention methods were highly effective. However, the level of depression and anxiety varied among the three groups. The effect of TD-GCBT and TD-CBT intervention approaches was better compared with the effect of TAU therapy, which was consistent with previous findings. Previous studies report that transdiagnostic CBT method has advantages over the use of conventional drugs in treatment of emotional disorders among the elderly (36). Hague et al. (11) used TD-CBT approach for treatment of a patient diagnosed with Major Depression and Generalized Anxiety Disorder. Alleviation of depression and anxiety symptoms was observed until the third month of follow-up. Marnoch (25) enrolled 16 elderly patients to a study and randomly assigned them to the TD-CBT group and delayed treatment group. The findings showed that the depression and anxiety scores of subjects in the treatment group were significantly lower after a 12-session intervention relative to the scores of subjects in the control group, indicating that TD-CBT method had high therapeutic efficacy. Alleviation of the symptoms may be because anxiety, depression and fear have common core risk factors or mediating mechanisms such as negative emotions and thought, stress, avoidance or withdrawn behavior (23). Transdiagnostic CBT reduces some maladaptive emotion regulation strategies such as meditation, worry and depression) and increases other more adaptive strategies including reassessment or distraction (37).

In this study, depression and anxiety scores among the elderly were alleviated even at nine months after the intervention (the 12th month), indicating a long-term therapeutic effect of the strategies. The TD-GCBT therapy exhibited the high efficacy, which is consistent with previous findings. For instance, a previous study comprising 1061 patients with emotional disorders was conducted to compare the effects of TD-GCBT plus TAU with TAU alone in Spain. The results indicated that TD-GCBT treatment alleviated emotional disorders at the 12th month after treatment of the subjects (38). During treatment, the subjects share their experience and help their peers with similar symptoms. Wuthrich conducted 11 sessions of group therapy over a 12-week period for elderly patients with depression and anxiety comorbidity. The results showed long-term therapeutic efficacy of the strategy (26). This effect is attributed to the patients’ continued activities after receiving intervention and learning the necessary effects. The elderly people are typically socially isolated and lonely as they lack important relationships, abilities, or vocations (39). Therefore, they can make some friends through communication and mutual help. The communication and socialization can help alleviate the symptoms, experience connection with the society, rediscover the meaning of life and improve their positive emotions (40–42).

However, TD-GCBT strategy has some limitations. First, TD-GCBT is mainly utilized for common emotional problems and cannot entirely alleviate all the symptoms in patients. Second, patients may feel insignificant since other members of the group have different symptoms. It is challenging to recognize and understand the symptoms and responses of other members (43). Third, therapists may not fully pay attention to all the disorders in each patient during the group intervention (44). Moreover, different patients have different levels of participation, which may affect their mastery and application of treatment information and technology. These factors may affect the outcome of the treatment. Group cohesion, positive group atmosphere, and the therapist’s positive attention to the neglected members in group psychotherapy are highly correlated with the treatment outcome (44). In addition, the therapist’s compliance with the operation manual, adherence to treatment and patient participation can affect the treatment results (45). Therefore, further studies should be conducted to assess the effect of these factors on the treatment outcome to improve the treatment efficacy (46).

The dropout rate in this study was significantly low, which may be attributed to many factors. First, we improved the protocol using the Delphi method to identify more appropriate treatment techniques for the Chinese elderly population. Additionally, we paid more attention to establishing positive relationships, including relationships between patient and therapist as well as within the patients. Mental health education and emotional support were provided to the patients during the treatment and follow-up process to make them comfortable. Second, China is characterized by Confucianism and the concept of filial piety, so patient autonomy is dictated by family values and doctor authority (47). The patients included in this study were recommended by the outpatient doctors and accompanied by their families. The patients had a high desire to be healed through psychotherapy, and their family members believed they would get better. Moreover, Anding Hospital is located in the center of Beijing; thus, there is effective transportation to the hospital. Patient compliance is an essential factor in determining treatment outcomes. A previous survey conducted from 2011 to 2014 revealed that the proportions of participants who reported loneliness, patients who had no one to talk to, and no one to help when it’s needed were 20.1, 4.21 and 10.5%, respectively (48). Chinese elderly patients require social interaction (49). The present study provided the elderly with social support and a platform to establish relationships with other people without incurring any cost.

The findings of the current study showed higher response rates compared with other similar studies. This can be attributed to the high overall education level of patients enrolled on the study and the moderate level of depression and anxiety, which ensured effective understanding of the treatment information and fostered change in their emotions. Moreover, this can be attributed to the inclusion of a higher proportion of female patients in the study. In addition, older women have a higher risk of loneliness than older men, and women readily share their negative emotions compared with men (50). Women can easily release old resentment by communicating with their peers with similar symptoms or experiences, ultimately improving their social and emotional well-being (51). Studies with larger clinical sample sizes and subjects with diverse severity and symptom manifestations should be conducted to further validate the effectiveness of the TD-GCBT approach.

## Limitations

The current study had some limitations. First, in our study, TD-GCBT and TD-CBT are non-inferior, while thee two strategies are superior to TAU. We did not find the best way to calculate the sample size for this study, so we referred to the sample size used in previous similar studies, and calculated it using G*power. In the future, we still need to explore more accurate sample size calculation methods to determine a more suitable sample size. Moreover, a high-quality multicenter RCT with a larger sample size should be conducted to verify the efficacy of the TD-GCBT strategy. Furthermore, the proportion of female patients in the final sample was 76.9% (90/117), which was higher compared with the proportion of male subjects. Studies should be conducted with equal number of both genders to explore the effect of gender factors on the treatment outcome.

## Conclusion

This is the first study to evaluate the efficacy of transdiagnostic cognitive behavioral group intervention for treatment of emotional disorders among the elderly with a 12-month follow-up in China. Depression and anxiety symptoms were alleviated after the intervention and up to 12-month follow-up. The efficacy of TD-GCBT and TD-CBT was similar, and the two strategies were more effective compared with the TAU approach. The findings from this trial provide a basis for the long-term efficacy of TD-GCBT for the treatment of emotional disorders among the elderly. The method used should be further improved in future, and RCT studies with larger sample sizes should be conducted to verify these findings.

## Data availability statement

The original contributions presented in this study are included in the article/supplementary material, further inquiries can be directed to the corresponding author.

## Ethics statement

The studies involving human participants were reviewed and approved by Beijing Anding Hospital Ethics Committee. Beijing Anding Hospital, Capital Medical University. The patients/participants provided their written informed consent to participate in this study.

## Author contributions

ZY contributed to the analysis and interpretation of data and drafting of this manuscript. FM developed the study design, critically reviewed, and revised the manuscript. MH recruited the patients, collected the data, and reviewed the manuscript. ZL conceptualized and designed the study, reviewed, and revised the manuscript. All authors contributed to the article and approved the submitted version.

## References

[1] Beard JR Officer AM Cassels AK. The world report on ageing and health. *Gerontologist.* (2016) 56(Suppl. 2):S163–6. 10.1093/geront/gnw037 26994257

[2] National Bureau of Statistics of China. *Main Data of the Seventh National Population Census.* Beijing: National Bureau of Statistics of China (2021).

[3] Härter MC. [Mental illness and physical disorders]. *Psychother Psychosom Med Psychol.* (2000) 50:274–86. 10.1055/s-2000-8822 10941287

[4] Saade YM Nicol G Lenze EJ Miller JP Yingling M Wetherell JL Comorbid anxiety in late-life depression: relationship with remission and suicidal ideation on venlafaxine treatment. *Depress Anxiety.* (2019) 36:1125–34. 10.1002/da.22964 31682328 PMC6891146

[5] Goldberg DP Krueger RF Andrews G Hobbs MJ. Emotional disorders: cluster 4 of the proposed meta-structure for DSM-V and ICD-11. *Psychol Med.* (2009) 39:2043–59. 10.1017/s0033291709990298 19796429

[6] Krueger RF Chentsova-Dutton YE Markon KE Goldberg D Ormel J. A cross-cultural study of the structure of comorbidity among common psychopathological syndromes in the general health care setting. *J Abnorm Psychol.* (2003) 112:437–47. 10.1037/0021-843x.112.3.437 12943022

[7] Casey LM Oei TP Newcombe PA. An integrated cognitive model of panic disorder: the role of positive and negative cognitions. *Clin Psychol Rev.* (2004) 24:529–55. 10.1016/j.cpr.2004.01.005 15325744

[8] Kube T Schwarting R Rozenkrantz L Glombiewski JA Rief W. Distorted cognitive processes in major depression: a predictive processing perspective. *Biol Psychiatry.* (2020) 87:388–98. 10.1016/j.biopsych.2019.07.017 31515055

[9] Reardon JM Williams NL. The specificity of cognitive vulnerabilities to emotional disorders: anxiety sensitivity, looming vulnerability and explanatory style. *J Anxiety Disord.* (2007) 21:625–43. 10.1016/j.janxdis.2006.09.013 17070666

[10] World Health Organization [WHO]. *Mental Health of Older Adults.* Geneva: WHO (2017).

[11] Hague B Scott S Kellett S. Transdiagnostic CBT treatment of co-morbid anxiety and depression in an older adult: single case experimental design. *Behav Cogn Psychother.* (2015) 43:119–24. 10.1017/s1352465814000411 25396319

[12] Lenze EJ Mulsant BH Shear MK Schulberg HC Dew MA Begley AE Comorbid anxiety disorders in depressed elderly patients. *Am J Psychiatry.* (2000) 157:722–8. 10.1176/appi.ajp.157.5.722 10784464

[13] Simning A Seplaki CL. Association of the cumulative burden of late-life anxiety and depressive symptoms with functional impairment. *Int J Geriatr Psychiatry.* (2020) 35:80–90. 10.1002/gps.5221 31650615 PMC6898755

[14] Smolders M Laurant M Verhaak P Prins M van Marwijk H Penninx B Adherence to evidence-based guidelines for depression and anxiety disorders is associated with recording of the diagnosis. *Gen Hosp Psychiatry.* (2009) 31:460–9. 10.1016/j.genhosppsych.2009.05.011 19703640

[15] Alexopoulos GS. Depression in the elderly. *Lancet.* (2005) 365:1961–70. 10.1016/s0140-6736(05)66665-2 15936426

[16] Boerner RJ. [Anxiety in elderly people – epidemiology, diagnostic features and therapeutic options]. *Fortschr Neurol Psychiatr.* (2004) 72:564–73. 10.1055/s-2004-818530 15472780

[17] Tham A Jonsson U Andersson G Söderlund A Allard P Bertilsson G. Efficacy and tolerability of antidepressants in people aged 65 years or older with major depressive disorder – a systematic review and a meta-analysis. *J Affect Disord.* (2016) 205:1–12. 27389296 10.1016/j.jad.2016.06.013

[18] Apóstolo J Bobrowicz-Campos E Rodrigues M Castro I Cardoso D. The effectiveness of non-pharmacological interventions in older adults with depressive disorders: a systematic review. *Int J Nurs Stud.* (2016) 58:59–70. 10.1016/j.ijnurstu.2016.02.006 27087298

[19] Andreescu C Varon D. New research on anxiety disorders in the elderly and an update on evidence-based treatments. *Curr Psychiatry Rep.* (2015) 17:53. 10.1007/s11920-015-0595-8 25980510

[20] Jonsson U Bertilsson G Allard P Gyllensvärd H Söderlund A Tham A Psychological treatment of depression in people aged 65 years and over: a systematic review of efficacy, safety, and cost-effectiveness. *PLoS One.* (2016) 11:e0160859. 10.1371/journal.pone.0160859 27537217 PMC4990289

[21] Palazzolo J. Cognitive behavioral therapy with depressed older adult: practical aspects and specificities. *Ann Med Psychol.* (2021) 179:21–6. 10.1016/j.amp.2020.07.003

[22] Seekles W Cuijpers P Kok R Beekman A van Marwijk H van Straten A. Psychological treatment of anxiety in primary care: a meta-analysis. *Psychol Med.* (2013) 43:351–61. 10.1017/s0033291712000670 22717105

[23] Fernández-Martínez I Orgilés M Morales A Espada JP Essau CA. One-Year follow-up effects of a cognitive behavior therapy-based transdiagnostic program for emotional problems in young children: a school-based cluster-randomized controlled trial. *J Affect Disord.* (2019) 262:258–66. 10.1016/j.jad.2019.11.002 31733917

[24] Bentley KH Bernstein EE Wallace B Mischoulon D. Treatment for anxiety and comorbid depressive disorders: transdiagnostic cognitive-behavioral strategies. *Psychiatr Ann.* (2021) 51:226–30. 10.3928/00485713-20210414-01 34433988 PMC8382208

[25] Marnoch SE. *A Pilot Randomised Controlled Trial Examining the Feasibility, Acceptability and Potential Efficacy of Transdiagnostic CBT for Depression and Anxiety in Older People.* Ph.D. thesis. London: king’s college london (2014).

[26] Wuthrich VM Rapee RM Kangas M Perini S. Randomized controlled trial of group cognitive behavioral therapy compared to a discussion group for co-morbid anxiety and depression in older adults. *Psychol Med.* (2016) 46:785–95. 10.1017/s0033291715002251 26498268

[27] Liu H Guo Z He M Li Z. A delphi study of transdiagnostic cognitive behavioral therapy techniques suitable for emotional disorders in the elderly. *J Clin Psychiatry.* (2020) 30:87–91.

[28] Geriatric Psychiatry Outreach Team. *The Change ways Geriatric Participant Manual.* Vancouver: Vancouver Coastal Health (2004).

[29] American Psychiatric Association [APA]. *Diagnostic and Statistical Manual of Mental Disorders (4th edn, text revision) (DSM-IV-TR).* 4th ed. Washington, DC: American Psychiatric Association (2001).

[30] Wang Z Zhang M. The application of mini-mental state examination (MMSE) in Chinese version. *Shanghai Arch Psychiatry.* (1989) 7:108–111.

[31] Hamilton M. A rating scale for depression. *J Neurol Neurosurg Psychiatry.* (1960) 23:56–62. 10.1136/jnnp.23.1.56 14399272 PMC495331

[32] Kroenke K Spitzer RL Williams JB. The PHQ-9: validity of a brief depression severity measure. *J Gen Intern Med.* (2001) 16:606–13. 10.1046/j.1525-1497.2001.016009606.x 11556941 PMC1495268

[33] Hamilton M. The assessment of anxiety states by rating. *Br J Med Psychol.* (1959) 32:50–5. 10.1111/j.2044-8341.1959.tb00467.x 13638508

[34] Spitzer RL Kroenke K Williams JB Löwe B. A brief measure for assessing generalized anxiety disorder: the GAD-7. *Arch Intern Med.* (2006) 166:1092–7. 10.1001/archinte.166.10.1092 16717171

[35] Liu X. The situation and path selection of China’s social support system for the elderly. *Popul Res.* (2012) 36:104–12.

[36] Muñoz-Navarro R Medrano LA Limonero JT González-Blanch C Moriana JA Ruiz-Rodríguez P The mediating role of emotion regulation in transdiagnostic cognitive behavioural therapy for emotional disorders in primary care: secondary analyses of the PsicAP randomized controlled trial. *J Affect Disord.* (2022) 303:206–15. 10.1016/j.jad.2022.01.029 34998804

[37] Sloan E Hall K Moulding R Bryce S Mildred H Staiger PK. Emotion regulation as a transdiagnostic treatment construct across anxiety, depression, substance, eating and borderline personality disorders: a systematic review. *Clin Psychol Rev.* (2017) 57:141–63. 10.1016/j.cpr.2017.09.002 28941927

[38] Cano-Vindel A Muñoz-Navarro R Moriana JA Ruiz-Rodríguez P Medrano LA González-Blanch C. Transdiagnostic group cognitive behavioural therapy for emotional disorders in primary care: the results of the PsicAP randomized controlled trial. *Psychol Med.* (2021) 8:1–13. 10.1017/S0033291720005498 PMC9772911 33550995

[39] Baladón L Fernández A Rubio-Valera M Cuevas-Esteban J Palao DJ Bellon JA Prevalence of mental disorders in non-demented elderly people in primary care [corrected]. *Int Psychogeriatr.* (2015) 27:757–68. 10.1017/s1041610214002841 25643982

[40] Agronin M. Group therapy in older adults. *Curr Psychiatry Rep.* (2009) 11:27. 10.1007/s11920-009-0005-1 19187705

[41] Floyd M Scogin F. Cognitive-behavior therapy for older adults: how does it work? *Psychotherapy.* (1998) 35:459–63.

[42] Tavares LR Barbosa MR. Efficacy of group psychotherapy for geriatric depression: a systematic review. *Arch Gerontol Geriatr.* (2018) 78:71–80. 10.1016/j.archger.2018.06.001 29933137

[43] Arnfred SM Aharoni R Hvenegaard M Poulsen S Bach B Arendt M Transdiagnostic group CBT vs. standard group CBT for depression, social anxiety disorder and agoraphobia/panic disorder: study protocol for a pragmatic, multicenter non-inferiority randomized controlled trial. *BMC Psychiatry.* (2017) 17:37. 10.1186/s12888-016-1175-0 28114915 PMC5260024

[44] Hyer L Yeager CA Hilton N Sacks A. Group, individual, and staff therapy: an efficient and effective cognitive behavioral therapy in long-term care. *Am J Alzheimers Dis Other Demen.* (2008) 23:528–39. 10.1177/1533317508323571 19001352 PMC10846160

[45] Zilcha-Mano S Snyder J Silberschatz G. The effect of congruence in patient and therapist alliance on patient’s symptomatic levels. *Psychother Res.* (2017) 27:371–80. 10.1080/10503307.2015.1126682 26837661

[46] Kazdin AE. Treatment outcomes, common factors, and continued neglect of mechanisms of change. *Clin Psychol.* (2005) 12:184–8. 10.1093/clipsy.bpi023

[47] Cheng SY Lin CP Chan HY Martina D Mori M Kim SH Advance care planning in Asian culture. *Jpn J Clin Oncol.* (2020) 50:976–89. 10.1093/jjco/hyaa131 32761078

[48] Zhang Y Hu W Feng Z. Social isolation and health outcomes among older people in China. *BMC Geriatr.* (2021) 21:721. 10.1186/s12877-021-02681-1 34922481 PMC8683828

[49] Chen Y Hicks A While AE. Loneliness and social support of older people living alone in a county of Shanghai, China. *Health Soc Care Community.* (2014) 22:429–38. 10.1111/hsc.12099 24621394

[50] Teh JKL Tey NP. Effects of selected leisure activities on preventing loneliness among older Chinese. *SSM Popul Health.* (2019) 9:100479. 10.1016/j.ssmph.2019.100479 31646167 PMC6804430

[51] Chan EA Lai CK. Understanding the reasons why Chinese older people do not wish to tell their life stories. *J Adv Nurs.* (2015) 71:1661–71. 10.1111/jan.12630 25656640

